# Loss of Merlin/NF2 protects pancreatic *β*-cells from apoptosis by inhibiting LATS2

**DOI:** 10.1038/cddis.2016.21

**Published:** 2016-02-18

**Authors:** T Yuan, K D D Gorrepati, K Maedler, A Ardestani

**Affiliations:** 1 Islet Biology Laboratory, Centre for Biomolecular Interactions Bremen, University of Bremen, Bremen, Germany; 2 German Center for Diabetes Research (DZD) project partner, University of Bremen, Bremen, Germany

*Dear Editor*,

A fundamental challenge in treating diabetes is the identification of molecular states that cause *β*-cell failure in response to pro-diabetic conditions. Both type 1 and type 2 diabetes mellitus result from an absolute or relative decline in pancreatic *β*-cell insulin secretion and/or mass. Apoptosis and loss of function are hallmarks of *β*-cell failure and the fundamental cause of diabetes.^1, 2^ We have recently identified mammalian sterile-20-like kinase (MST1), the key component of Hippo signaling, as a novel regulator of pancreatic *β*-cell death and dysfunction in human and rodent *β*-cells *in vitro* as well as in diabetic animal models *in vivo*. MST1 promotes *β*-cell apoptosis through regulation of multiple downstream targets, such as c-Jun N-terminale Kinase (JNK), caspase-3, histone H2B and mitochondrial Bcl-2 member family.^3^ However, the mechanism of upstream regulation of MST1 in the *β*-cell and the ‘*β*-cell Hippo signaling' cascade has not yet been investigated. Such critical upstream regulator of the Hippo signaling pathway is neurofibromatosis type 2 (NF2) or Merlin, a tumor suppressor protein, which belongs to the ezrin–radixin–moesin family of actin-binding proteins. It is conserved in both Drosophila and mammals, and plays a key role in organ-size control and development through the regulation of cell proliferation and apoptosis.^4^ NF2 initiates the Hippo signaling by directly activating MST1/2 kinases,^5^ or by recruiting large-tumor suppressor kinase 1/2 (LATS1/2) to membranes for phosphorylation by MST1/2 without altering intrinsic MST1/2 kinase activities.^6^ So far, the physiological role of NF2 in the *β*-cell, and whether its loss would regulate *β*-cell death and insulin secretion as well as downstream Hippo kinases are not known.

NF2 is expressed in isolated human islets, in the rat insulinoma *β*-cell line INS-1E (Figure 1) as well as in mouse islets (data not shown). Inhibition of endogenous NF2 by siRNA knockdown rescued INS-1E cells from high glucose- and high-glucose/palmitate-induced apoptosis, as demonstrated by decreased caspase-3 levels and Poly-(ADP-ribose)-polymerase (PARP) cleavage (Figure 1a). Consistently, NF2 silencing protected *β*-cells from pro-inflammatory cytokines and high-glucose/palmitate-induced apoptosis in isolated human islets (Figure 1a). As NF2 functions upstream of the core Hippo pathway kinase LATS1/2 and MST1/2, we aimed to identify whether NF2 changes LATS1/2 and MST1/2 activities under diabetic conditions. Although NF2 knockdown did not change MST1 hyperactivation under glucotoxic conditions, it remarkably reduced LATS1/2 phosphorylation demonstrating NF2-dependent LATS1/2, but not MST1/2 regulation in *β*-cells (Figure 1a). This is in line with the recently suggested alternative model of NF2 function through direct binding to LATS proteins.^6^ Intriguingly, LATS2 reconstitution followed by NF2 knockdown in INS-1E cells abrogated *β*-cell protection by NF2 loss, confirming LATS2-dependent action of NF2 in pancreatic *β*-cells (Figure 1a).

NF2 controls cell survival by integrating signals initiated through cell–cell interactions or extracellular cues by direct suppression of growth regulatory and antiapoptotic pathways, including the mechanistic target of rapamycin (mTOR) signaling.^7^ As mTORC1 is a critical pro-survival signal in *β*-cells whose transient hyperactivation has pleiotropic functions leading to increased *β*-cell mass,^8^ we checked whether NF2 loss has an impact on mTORC1 activity, as represented by downstream substrates S6K and 4EBP1 phosphorylation. Notably, S6K as well as 4EBP1 phosphorylation was highly upregulated by NF2 depletion in both isolated human islets and INS-1E cells, suggesting direct regulation of *β*-cell antiapoptotic mTORC1 by NF2 (Figure 1b). Despite its critical role in apoptosis inhibition, NF2 depletion neither affects glucose-stimulated insulin secretion, nor insulin gene expression and critical genes involved in glucose sensing and insulin transcription. Thus, NF2-dependent *β*-cell protection occurred without affecting *β*-cell function (Figure 1c).

In conclusion, our data show a direct protective effect of NF2 depletion in pancreatic *β*-cells by inhibiting LATS2 but not MST1 activity, which could rescue *β*-cells from apoptosis without compromising *β*-cell function. Also, mTORC1 hyperactivation might be involved in the pro-survival mechanism of NF2 deficiency. The identification of NF2 as the key upstream regulatory and disease-relevant component of the Hippo signaling provides a novel area for potential therapeutic approaches aiming to block *β*-cell apoptosis in order to restore a functional pancreatic *β*-cell mass in diabetes.

## Figures and Tables

**Figure 1:**
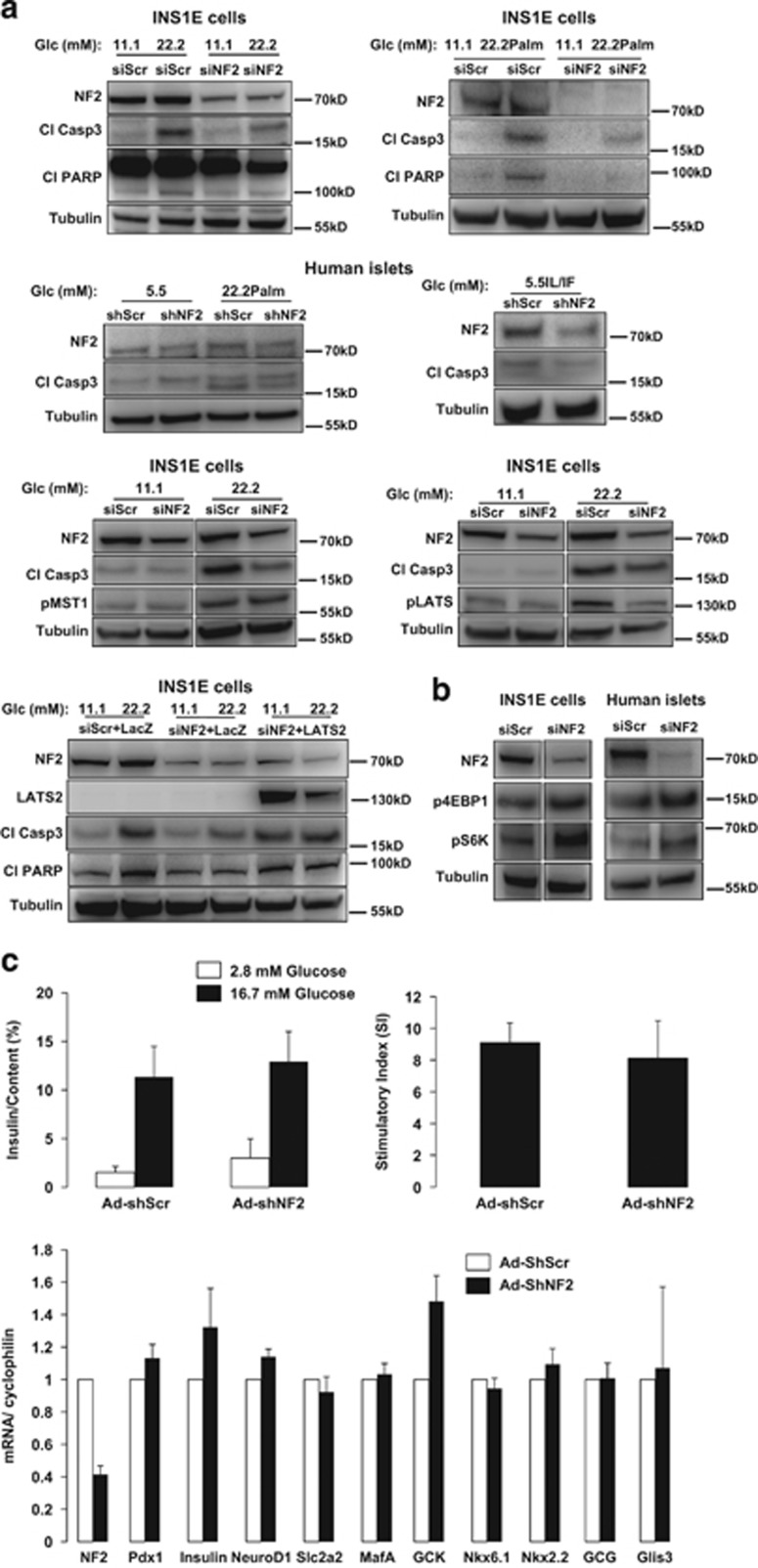
(**a**) INS-1E cells were transfected with either control Scr siRNA (siScr) or NF2 siRNA (siNF2) (both from Dharmacon, Lafayette, CO, USA) and treated with 22.2 mM glucose or the mixture of 22.2 mM glucose and 0.5 mM palmitate (22.2  Palm) for 2 days. Human islets were infected with either control Ad-GFP-Scr or Ad-GFP-hShNF2 adenoviruses (VectorBioLabs, Malvern, PA, USA) for 1 day and then treated with the mixture of 22.2 mM glucose and 0.5 mM palmitate or the pro-inflammatory cytokines 2 ng/ml recombinant human IL-1*β* and 1000  U/ml IFN*γ* (IL/IF) for 3 more days. INS-1E cells were transfected with either control siScr or siNF2 and treated with 22.2 mM glucose for 2 days. INS-1E cells were transfected with either control siScr or siNF2 for 1 day and then infected with either Ad-LacZ control or Ad-LATS2 adenoviruses (VectorBiolab) and then treated with 22.2 mM glucose for another day. (**b**) INS-1E cells and isolated human islets were transfected with either control siScr or siNF2. Cleaved capase-3, cleaved PARP, NF2, pMST1, pLATS1/2, LATS2, pS6K and p4EBP1 were analyzed by western blotting. Western blots show representative results from three independent experiments (INS-1E) from three different donors (human islets). Tubulin was used as loading control. (**c**) Human islets were infected with either control Ad-GFP-Scr or Ad-GFP-hshNF2 adenoviruses (VectorBiolab) for 2 days. Insulin secretion during 1 h incubation with 2.8 mM (basal) and 16.7 mM glucose (stimulated), normalized to insulin content. The insulin stimulatory index denotes the ratio of secreted insulin during 1 h incubation with 16.7 and 2.8 mM glucose. Pooled data from three independent experiments from three different donors (human islets). Quantitative RT-PCR for *NF2, Insulin, Pdx1, NeuroD1, Slc2a2, MafA, GCK, Nkx6.1, Nkx2.2, GCG and Glis3*. For analysis, we used the AppliedBiosystems StepOne Real-Time PCR system (Applied Biosystems, Carlsbad, CA, USA) with TaqMan(R) Fast Universal PCR Master Mix for TaqMan assays (AppliedBiosystems). Pooled data from four independent experiments from four different donors (human islets)
